# The Ogden model of rubber mechanics: 50 years of impact on nonlinear elasticity

**DOI:** 10.1098/rsta.2021.0332

**Published:** 2022-10-17

**Authors:** Michel Destrade, Luis Dorfmann, Giuseppe Saccomandi

**Affiliations:** ^1^ School of Mathematical and Statistical Sciences, NUI Galway, Galway, Republic of Ireland; ^2^ Department of Civil and Environmental Engineering, Tufts University, Medford, MA 02155, USA; ^3^ Dipartimento di Ingegneria, Università degli studi di Perugia, 06125 Perugia, Italy

**Keywords:** rubber mechanics, elasticity, Ogden model

## Abstract

We place the Ogden model of rubber elasticity, published in *Proceedings of the Royal Society* 50 years ago, in the wider context of the theory of nonlinear elasticity. We then follow with a short interview of Ray Ogden FRS and introduce the papers collected for this Theme Issue.

This article is part of the theme issue ‘The Ogden model of rubber mechanics: Fifty years of impact on nonlinear elasticity’.

## 1. The context: nonlinear elasticity theory for rubber

When we examine the development of the theory of rubber-like elasticity through the ages, we may identify a divide from protohistory to history by singling out the 1940 landmark paper by Mooney [1]. And so, when in 1972, the *Ogden model* [2] appeared on the scene, more than 30 years had already passed in attempting to construct a strain-energy function capable of being both descriptive and predictive of the observed experimental behaviour of rubber-like solids. In the opinion of Ray Ogden, ‘[in these attempts] it was convenient and practical to use certain stretch or strain invariants as independent variables in preference to the principal stretches’. That was the approach pioneered and championed by Rivlin. To understand how the Ogden model came about, we first propose a brief survey of these various attempts.

As recalled in Treloar’s book [3], Kelvin Kuhn (in 1936) and Eugene Guth (in 1939) used a kinetic theory, based on the Gaussian statistics for macromolecular chains and network theory, to model the response of rubber. This resulted in the *neo-Hookean model* for W, the strain energy density function

1.1 $$W=\frac{\mu }{2}(I_{1}-3),$$

where μ is the shear modulus and $I_{1}=\text{tr}\;C$ is the first principal strain invariant (here $C=F^{T}F$ and F is the gradient of the deformation). These authors used the principal stretches $\lambda_{1},\lambda_{2}$, $\lambda_{3}$ of the deformation via the *affine deformation assumption* to relate the macroscopic deformation to the individual chains composing the network.

In 1940, Mooney’s [1] starting point was based on three basic assumptions: (i) the material is isotropic, (ii) the deformation is isochoric and (iii) the traction in simple shear in any isotropic plane is proportional to the amount of shear. Working in terms of the principal stretches, he derived the following strain energy density,

1.2 $$W(\lambda_{1},\lambda_{2},\lambda_{3})=C_{1}(\lambda_{1}^{2}+\lambda_{2}^{2}+\lambda_{3}^{2}-3)+C_{2}(\lambda_{1}^{-2}+\lambda_{2}^{-2}+\lambda_{3}^{-2}-3),$$

where $C_{1}$ and $C_{2}$ are two constants. Clearly (1.3) may be recast in the form

1.3 $$W(I_{1},I_{2})=C_{1}(I_{1}-3)+C_{2}(I_{2}-3),$$

where $I_{2}=[I_{1}^{2}-\text{tr}\;C^{2}]/2$ is the second principal strain invariant. It is worth noting that Mooney never mentioned the term ‘invariants’.

In fact, it was Rivlin who used the principal invariants in a systematic and consistent way, with a series of papers published in the *Philosophical Transactions of the Royal Society* beginning in 1948, see [4] for an historical overview of these works. The strain energy density function (1.4) is now referred to as the *Mooney–Rivlin model*. Starting with Rivlin, the use of the full methodological apparatus of linear algebra became fundamental to the development of the modern theory of nonlinear continuum mechanics, as is attested, for example, by the ubiquitous role of the Hamilton–Cayley theorem in constitutive modelling.

In 1944, Treloar performed a series of experiments on rubber [5], which to this day are still used as a benchmark set of data. Comparing the predictions of (1.2) with his data, he noted on page 99 of his book [3],

The conclusion to be drawn from the experimental observations […] is that the formulae of the statistical theory, involving a single physical constant, correctly describe the properties of a real rubber to a first approximation. […] However, […] it is not surprising that some deviations from the ideal theoretical behaviour are to be found.

Although it was erroneous to conclude that the neo-Hookean model is good in a certain range of deformation (as explained in [6], for instance), that statement indicated that modelling the *deviation* using statistical theory is challenging and, as a result, stimulated much transformative research in rubber-like mechanics.

In 1951, Rivlin & Saunders [7] proposed an extension of (1.3) to address this issue. Specifically, they proposed an energy function of the form

1.4 $$W(I_{1},I_{2})=C_{1}(I_{1}-3)+f(I_{2}-3),$$

where f is an unknown function to be determined from experiments. Although Rivlin often restricted attention to a linear function f, as in (1.3), he nonetheless accelerated the search of a specific form of such a function. An overview of the different forms of f proposed during this period is given by Hart–Smith [8], see also the notable paper by Gent & Thomas [9].

In the Introduction to his 1972 paper, Ogden [2] writes

However, such choices of independent variable in general needlessly complicate the associated mathematical analysis. […] Principal axes techniques […] obviate the need for any special choice of invariants and, moreover, by use of such techniques, the basic elegance and simplicity of isotropic elasticity is underlined.

Ogden considered a strain energy density function in the form $W=W(\lambda_{i},\lambda_{j},\lambda_{k})$, where i≠j≠k. A requirement connected to this form is that for isotropic materials, W remains invariant for any permutation of the indices i,j,k. To overcome this problem, Carmichael & Holdaway [10] and then Valanis & Landen [11] proposed the form

1.5 $$W(\lambda_{1},\lambda_{2},\lambda_{3})=\varphi (\lambda_{1}^{2})+\varphi (\lambda_{2}^{2})+\varphi (\lambda_{3}^{2})-3\varphi (1),$$

where φ is an unknown function to be determined from experiments. The choice of (1.5) is *heuristic* in nature, and indeed no justification of this form was proposed other than its inherent mathematical simplicity to satisfy the symmetry conditions imposed by isotropy. Carmichael & Holdaway [10] developed an explicit form of φ based on the first two assumptions of Mooney and an additional complicated assumption on the stress–strain relation in simple shear. By contrast, Valanis & Landen [11] proposed the following simpler form:

1.6 $$\varphi (\lambda_{i})=2\mu \lambda_{i}(\log\lambda_{i}-1),$$

a choice that was entirely empirical.

The basis of the Ogden model is more rational in many respects. Its starting point can be traced to the paper by Hill [12], which introduces the family of Lagrangian strain measures

1.7 $$e_{i}=\frac{\left(\lambda_{i}^{\alpha }-1\right)}{\alpha },$$

for α≠0, and $e_{i}=\log\lambda_{i}$ in the α=0 limit. Defining a strain measure in terms of the stretches allows to write the incremental work dW as a linear combination of the components of the differential strain de,

1.8 dW=τde,

where τ is the current stress. In (1.7), to each value of α corresponds a conjugate measure of stress such that dW is invariant. For example, the Biot stress tensor and the second Piola–Kirchhoff stress tensor are the conjugates of $\vec{e}$ when α=1,2, respectively [12].

Then, for α≠0, Ogden introduced the first principal invariant of (1.7) as

1.9 $$\phi (\alpha )=\frac{1}{\alpha }(\lambda_{1}^{\alpha }+\lambda_{2}^{\alpha }+\lambda_{3}^{\alpha }-3),$$

and considered the functional form

1.10 $$W=\sum_{r}\mu_{r}\phi (\alpha_{r})=\sum_{r}\frac{\mu_{r}}{\alpha_{r}}(\lambda_{1}^{\alpha_{r}}+\lambda_{2}^{\alpha_{r}}+\lambda_{3}^{\alpha_{r}}-3).$$

He showed that the quantity $\sum \mu_{r}\alpha_{r}/2$ gives the initial shear modulus (which must be positive), and that $\alpha_{r}$ may be allowed to be a non-integer. This facilitates the correlation with experimental data and permits a very good fit to the data with a small number of terms.

**Figure 1.**
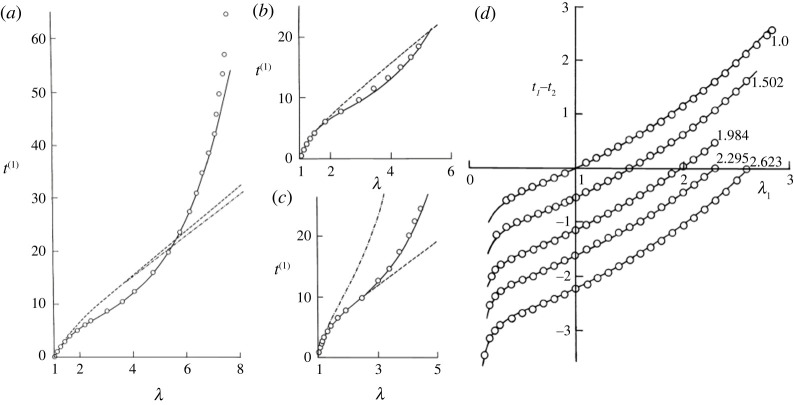
Performance of the three-term (six parameters) Ogden model (continuous curves) at modelling the mechanical response of rubber (circles) [13]. (*a*–*c*) Fitting the 1944 data of Treloar [5], (*a*) of simple tension, (*b*) of pure shear and (*c*) of equibiaxial tension. Comparisons with the new-Hookean and Mooney–Rivlin models (broken lines) are shown. Image (*d*) illustrates the Valanis–Landen requirement [11] of data translation in biaxial experiments; the experimental data are from Jones & Treloar [14] with $\lambda_{2}$ fixed at 1.0, 1.502, 1.984, 2.295, 2.623. (Adapted from [13], with permission from Dover Publishing.)

A notable advantage of the model (1.11) is that the number of fitting constants can be increased as desired to improve the correlation with experimental data, see figure 1. Notably, the Rivlin expansion of W,

1.11 $$W(I_{1},I_{2})=\sum_{m,n=0}^{\infty }C_{mn}{\left(I_{1}-3\right)}^{m}{\left(I_{2}-3\right)}^{n},$$

also has a similar advantage, but differs in one important point when compared with the Ogden model. Indeed, any analytic strain energy density function $W(I_{1},I_{2})$ has a Taylor expansion in the form (1.11), but Taylor series are an accurate approximation only locally, i.e. around the unstrained reference configuration. By contrast, the Ogden model (1.10) is not a Taylor expansion.

Another fundamental property of the model (1.11) is highlighted in the book by Ogden [13]. When we consider the experimental data of a *biaxial* deformation of a rectangular sheet, the Cauchy principal stresses are given by

1.12 $$t_{i}=\lambda_{i}\frac{\partial W}{\partial \lambda_{i}}-p,\;i=1,2,3,$$

where p is the Lagrangian multiplier associated with the incompressibility constraint. Depicting $t_{1}-t_{2}$ against $\lambda_{1}$ at constant $\lambda_{2}$, we find that the data possess a shape-invariant property. Specifically, the data curves for different values of $\lambda_{2}$ may be superposed by a vertical translation. That property is captured by any W of the form (1.5) and thus, by the Ogden model, see figure 1*d*.

Over the years, many works have referred to the Ogden model, including patents, finite-element software codes and more than 3600 journal articles citing the original 1972 paper [2] and 6000 citing the related textbook [13]. Here we highlight the results of three papers.

The first, by Ogden [15], shows that for incompressible solids, each principal component of the distortional part of the stress can be expressed as a function of the corresponding principal component of strain only, up to the fourth order in the strain. This result may be used to justify the separability hypothesis (1.5), up to a certain order at least.

Twizell & Ogden [16] present a systematic optimization procedure to investigate the correlation between theory and experiments. The numerical procedure proposed improves upon existing methods that were used to determine the material constants ($\mu_{r}$, $\alpha_{r}$) as best-fit parameters. The authors also found that the correlation between the data and the model is casual or in some sense, spurious. This is explained by the fact that the Ogden model is purely phenomenological and does not connect the material constants to mesoscopic quantities or mechanical features.

As shown later by Ogden *et al.* [17], the curve-fitting procedure of (1.10) is a *nonlinear optimization problem* and its solution is not unique for n≥2. Hence, several combinations of material parameters exist that provide the same level of optimal fitting, but with the predictions of the corresponding models being quantitatively different, sometimes widely. This problem is a widely acknowledged limitation of the Ogden model.

## 2. An interview with Ray Ogden FRS

**Figure:**
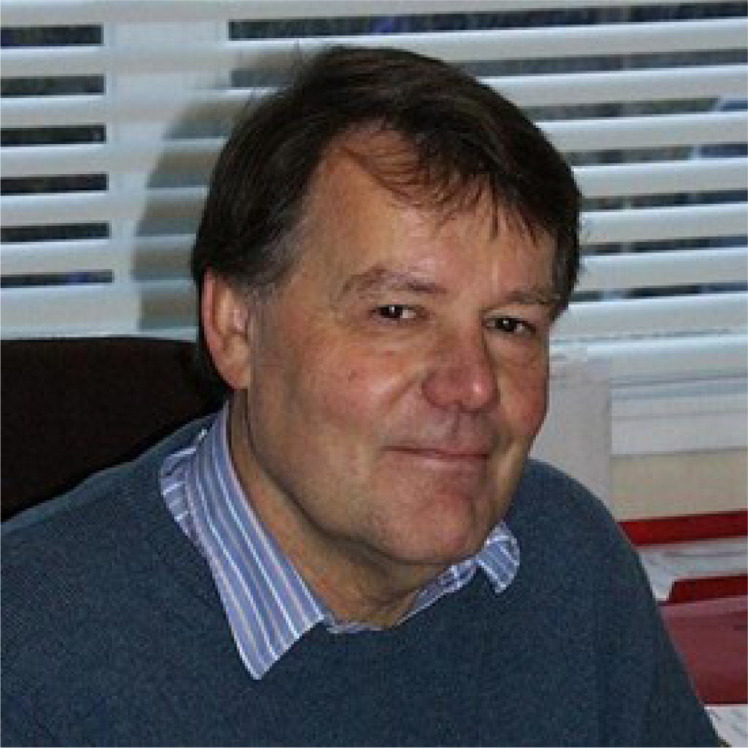

**What was the status of solid mechanics in the UK in the 1970s?**

The solid mechanics community was actually quite strong in the 1970s. I can think of at least 40 names of people working in solid mechanics, with groups in East Anglia, Nottingham, Oxford, Cambridge, Manchester, Sheffield, etc. It was a relatively large community, but nowhere near as large as the fluid mechanics community.

**Did you develop relationships with Rivlin, Truesdell, Knowles, Ericksen, etc., in the USA? And were there people in Europe connected to you?**

I never met Truesdell, but I used his volume in the Handbuch der Physik as my bible when I was a PhD student. I met Rivlin, Knowles, Ericksen, Sternberg, Wineman, etc. As far as I know, they didn’t have big groups. In continental Europe there was very little activity in nonlinear elasticity then.

**Do you remember the starting point of your model? How did it fit within the developments in nonlinear elasticity at the time?**

I started working on it towards the end of my PhD when Rodney Hill suggested using stretches in the strain energy function. This hadn’t been used a great deal except possibly for the Valanis–Landen model. People were mostly using invariants at that point.

**Was there a connection between you and Treloar?**

I visited him in Manchester and he gave me a lot of data from his experiments. That data were the basis for fitting the model originally.

**It must have been hard to perform the curve-fitting at that time. You didn’t use a computer?**

Right! I had to do it by hand, basically. I tried to fit the lower part of the curve with one term, and I took a second term to fit the upper part. I adjusted the numbers so that they fit both parts of the curve but then, it needed a third term so that the whole range of the data was fitted for simple tension, for example.

**What happened just after the model was published, do you remember the early reactions?**

I’m not sure that I encountered much reaction at the time! It took off very slowly, and it took many years to actually build up until it was widely used.

**The model has been implemented in almost all finite-element codes of solid mechanics. Were you ever approached to discuss those implementations?**

No, the developers just did it on their own accord, without consulting me.

**Was it unusual for someone in a Mathematics department to be interested in fitting experimental data?**

I think it probably was. In fluid dynamics it wasn’t quite so unusual because some mathematicians were actually doing experiments. But nowadays, more people in mathematics departments are doing experiments in solid mechanics like for example, experiments on moving microorganisms.

**What’s your take on your model today?**

I’m very gratified that it became a highly cited model, widely used, and this is partly because it’s been implemented in various commercial software codes. But the model is only an isotropic model. Nowadays, there’s much more emphasis on anisotropic materials in the context mainly of biological tissues, …, but that’s another story!

## 3. Contents of the Theme Issue

With this volume, we show that the ideas underlying the Ogden model are alive and well and that the model is essential for the ongoing developments of nonlinear elasticity. Its application has had a transformative impact on the use of the nonlinear theory of elasticity in the design of new components and devices, has generated a wealth of new information and has improved and deepened our understanding of the large deformation behaviour of soft matter. The papers presented here provide the reader with an, albeit partial, overview on the broad use of the celebrated Ogden material model, published exactly 50 years ago in *Proceedings of the Royal Society*.

Anssari-Benam *et al*. [18] discuss constitutive models that characterize the hyperelastic response of brain tissue subject to mechanical stimuli. They find that single- or multiple-term Ogden models can result in unsatisfactory numerical results when modelling the extremely soft and heterogeneous brain tissue. They propose strain energy functions (generalized neo-Hookean models, modified Ogden model) yielding more accurate numerical results.

Ciambella *et al*. [19] exploit the polyconvexity of the Ogden model to derive a model for the progressive reduction of material stiffness resulting in cohesive failure of elastomeric materials at large strain. They define a degradation function to quantify the elastic energy reduction due to damage. Their model is applicable to fracture coalescence and damage propagation in a wide range of materials.

Ehret & Stracuzzi [20] use the molecular statistical theory of rubber elasticity to present the Ogden model in terms of the non-affine three-chain theory of non-Gaussian chains. They recover well-known hyperelastic models and obtain new nonlinear elastic energy functions able to describe the behaviour of rubber-like materials.

Guo *et al.* [21] study the bulging of a rubber tube with fixed ends when it is inflated by internal pressure. Deriving the bifurcation condition for localized bulging is an involved mathematical feat. It is a highly nonlinear process, with strong dependence on the model chosen for the material. Here the authors show that the Ogden model predicts a different bulging behaviour than the Gent and Gent-Gent [17] models. They also perform experiments on tubes made of natural latex rubber.

Horgan & Murphy [22] discuss the use of one-term Ogden models to predict the responses of both incompressible elastomers and soft tissues. They argue that model parameters may be found to give excellent agreements with some aspects of the mechanical response, but may not be physically realistic in other situations. Hence, the predictions of models with either negative or large positive exponents do not seem physically realistic in simple shear.

Kaliske *et al*. [23] take the Ogden Law as the starting point to derive a rate-dependent model for quasi-incompressible electroactive materials. They use the principle of virtual power to derive a mixed finite-element formulation of an electromechanical phase-field fracture model. They perform finite strain experiments on the dielectric material VHB 4905^TM^ to identify the material parameters of their modified Ogden model.

Lohr *et al*. [24] discuss the relevance of Ogden’s model to characterize the mechanical properties of soft tissues. They use pure shear data of brain tissue and blood clots for the model parameter identification of a one-term Ogden form. They make a connection with the polymer chain and network theory reported by Ehret & Stracuzzi [20].

In 1979, Ogden & Haughton [25] derived the bifurcation conditions for a circular cylindrical tube of an elastic material subject to combined axial load and internal pressure. Melnikov *et al*. [26] expand the theory to account for residual-stress and a radial electric field. Here, axisymmetric incremental deformations combined with increments in the electric displacement are superimposed on a known finitely deformed configuration. The governing equations and boundary conditions are first obtained in general form and then specialized for the neo-Hookean and Ogden electroelastic models.

Menzel & Witt [27] aim to provide an improved understanding of the electromechanical coupling phenomenon and extremal states in large deformation electroelasticity. Specifically, they analyse the change in stress resulting from changes in the electric field. This connection is governed by the third-order electroelastic tensorial moduli, which are not constant but depend on deformation and electric field. The authors also propose visualization tools for the third-order tensors.

Mihai *et al*. [28] focus on the nonlinear response of nematic liquid crystal elastomers. They note that in uniaxial tensile tests, the material sample does not contract in the direction perpendicular to the applied load, but expands for sufficiently large tensile strains, while its volume remains unchanged. Motivated by this response, they propose an Ogden-type strain-energy function and use experimental data to calibrate the material parameters. They find that Ogden strain-energy functions are particularly suitable for modelling nematic elastomers because of their mathematical simplicity.

Nikolov *et al*. [29] propose a novel identification method for the Ogden material parameters. In this method, the fully three-dimensional displacement field of the mapping function between a reference and deformed configuration as well as the corresponding loads are measured concurrently. The authors provide a method that leverages the weak-form of the boundary value problem to effectively use full-field, heterogeneous deformation data extracted from the experiments.

Saccomandi *et al*. [30] show that using the fourth-order weakly nonlinear theory of elasticity results in an improved estimate of the material stiffness of healthy and diseases tissues compared with the fully nonlinear theory. The authors discuss in detail some of the shortcomings encountered by some sophisticated models developed within the latter theory when describing the responses of real materials.

Selvadurai [31] analyses the mechanics of deformation of incompressible planar hyperelastic membranes, rigidly fixed at their boundaries and subject to uniform pressure. He focuses on the neo-Hookean, Mooney–Rivlin and Ogden strain energy forms, and solves the governing equations numerically using the finite element method. Of particular interest is the wrinkling instability observed in membranes of plane circular and elliptical forms.

Finally, Yao *et al*. [32] devise a method to derive the volumetric part of the strain energy function, so long as the incompressible strain energy term is given. The method is used to obtain a generalized Ogden model for compressible rubber-like materials.

## 4. Concluding remarks

A famous aphorism by George Box [33] says that ‘essentially, all models are wrong, but some are useful’. We now know that in the theory of nonlinear elasticity, the quest for ‘the’ strain-energy function has been a chimaera and we recognize that the approximate nature of the Ogden model must always be kept in mind.

The Ogden model has been fundamental to the advancement of nonlinear elasticity theory in several principal directions, including, but not limited to, the following:

— The Ogden model, together with the earlier Mooney–Rivlin model, provides clear evidence that an approach based on a rigorous mathematical foundation is superior to an empirical approach, the latter qualified by Ericksen as the ‘somewhat mystical process whereby we detect definite forms of constitutive equations’ [34].
— With the Ogden model, it became apparent that no set of invariants is intrinsically superior to another in modelling data.
— The Ogden model shows that data of rubber can be fit to a desired accuracy in a systematic way.
— The Ogden model is able to describe reasonably the experimental data of various soft materials, a robustness that is needed for efficient implementation into any computer simulation model.
— The Ogden model has been a catalyst to extend nonlinear mechanics to other frameworks beyond pure nonlinear elasticity and include coupled fields theories.

Last but not least, the model has acknowledged limitations, but addressing them has proved fundamental to sustain the development of the theory of nonlinear elasticity and its applications. Again in the words of George Box, ‘Since all models are wrong the scientist must be alert to what is importantly wrong. It is inappropriate to be concerned about mice when there are tigers abroad’.

## Data Availability

This article has no additional data.
